# Supplementary material for: Genomic skimming and nanopore sequencing uncover cryptic hybridization in one of world’s most threatened primates
Source: Sci Rep. 2021 Aug 26;11:17279. doi: 10.1038/s41598-021-96404-6 (PMC8390465; doi:10.1038/s41598-021-96404-6)
Supplement: Supplementary file 1 — Supplementary Information. [file 41598_2021_96404_MOESM1_ESM.pdf]

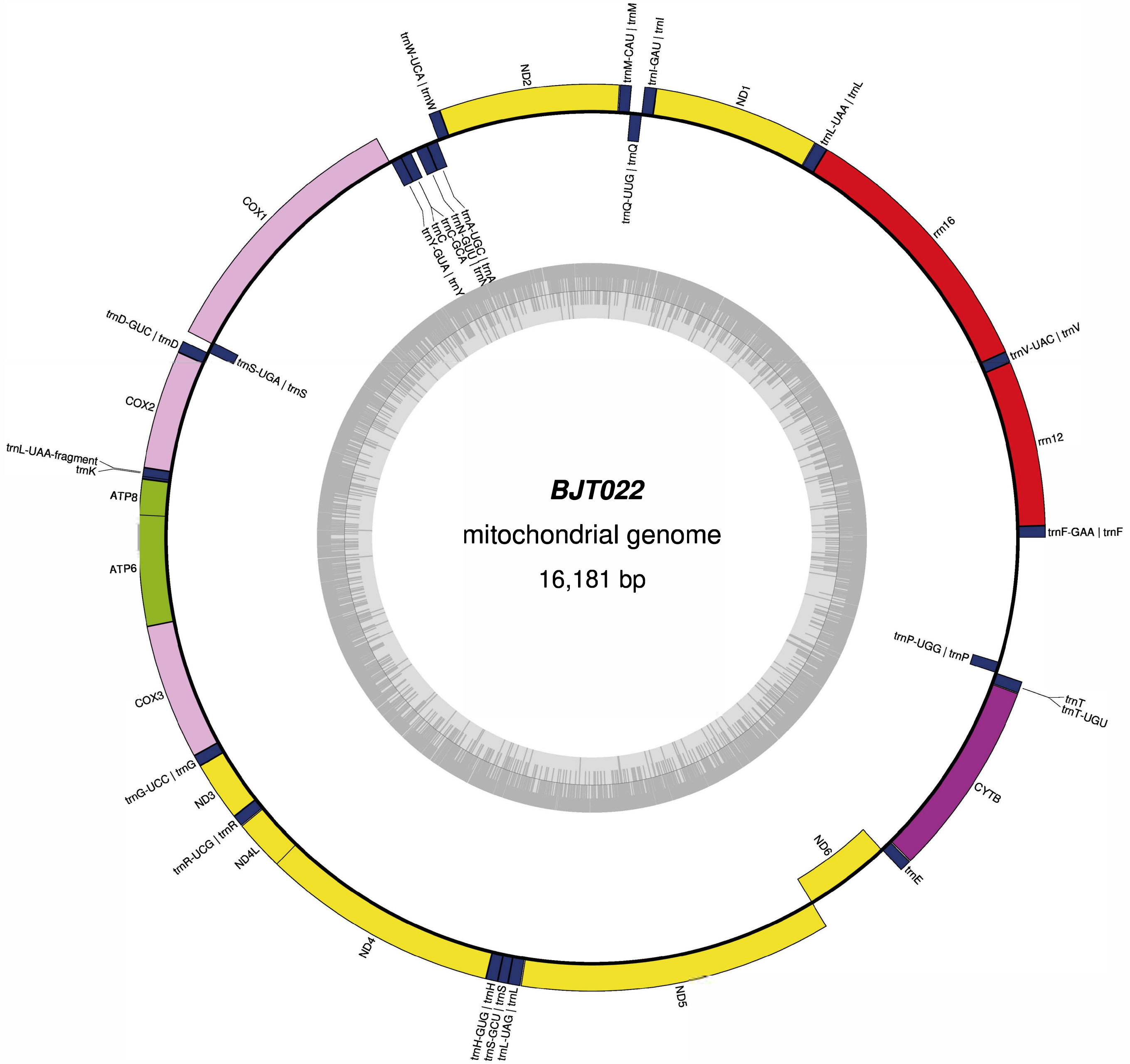

- complex I (NADH dehydrogenase)
- complex IV (cytochrome c oxidase)
- ATP synthase
- other genes
- transfer RNAs
- ribosomal RNAs

Supplementary Figure 1. Circular map of the marmoset mitogenome obtained from ONT sequencing. Color legend in bottom left corner indicates annotated features of the mitogenome assembly, and the inner circle within the plot shows sequence %GC content. This map was produced as part of mitogenome annotation we performed on the CHLOROBOX web server (https://chlorobox.mpimp-golm.mpg.de),

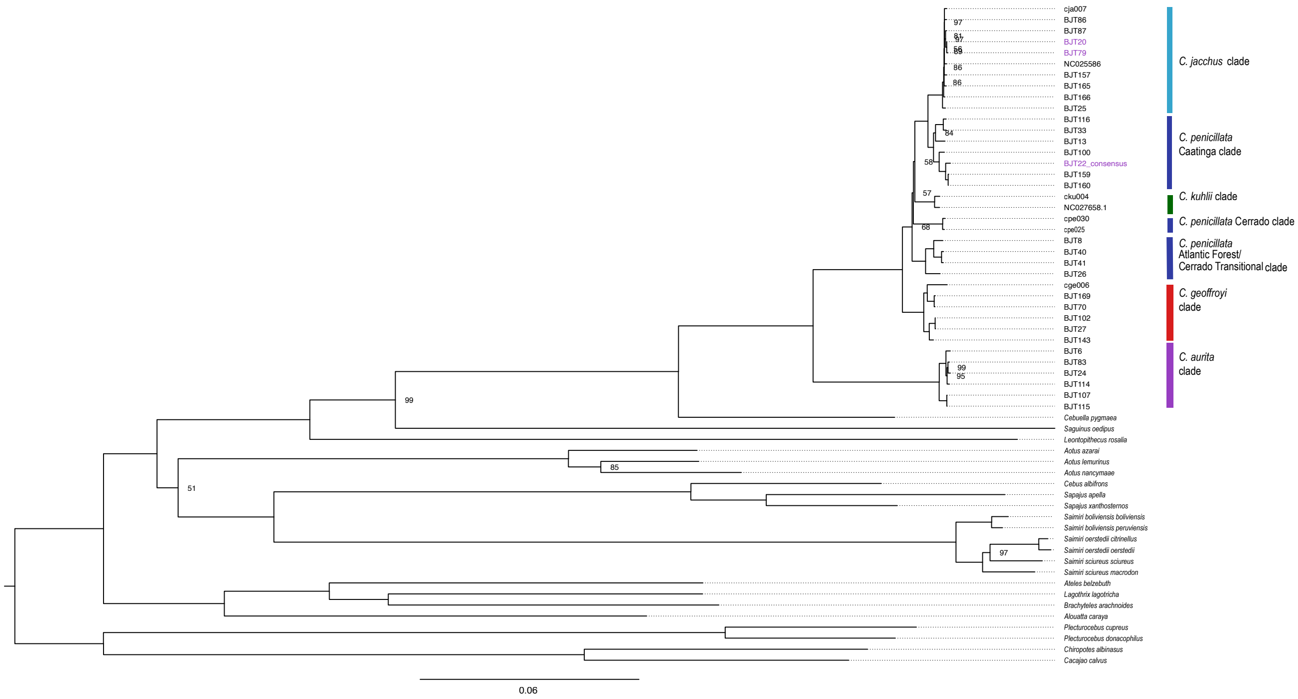

Supplementary Figure 2. ML tree showing phylogenetic clustering of the *Callithrix* mitogenome of marmoset BJT022 within the *C. penicillata* Caatinga clade. The BJT022 mitogenome is highlighted at the tree tips in purple along with two other mitogenomes of marmosets with *C. aurita* phenotypes from Malukiewicz et al. (2021)[1] that were incongruent with their mitogenomic lineages. Categorization of major *Callithrix* mitogenomic clades follow that of Malukiewicz et al. (2021)[1].

Table S1. Sample metadata for newly generated *Callithrix* mitogenome from individual BTJ022 and mitogenomic sequences used from Malukiewiczet al. (2021)[1]. The 'Sample' column gives ID of each new sampled individual or the species for sequences obtained from previous studies. The 'Accession' column gives Genbank accession numbers for each sequence. The 'Phenotype' column indicates whether the sampled individual possessed a pure species or hybrid phenotype, and capital letters in parentheses next to *Callithrix penicillata* x *Callithrix geoffroyi* category are specific phenotype classifications following Figure 5 in Fuzessy et al. (2014)[2]. The 'mtDNA Lineage' column indicates phylogenetic classification of the mitochondrial genome of the sampled individual. The 'Sampling Location' column indicates where each individual was sampled. Nearest cities are located for individuals sampled from the wild, and facilities are indicated for individuals sampled in captivity. The Guarulhos Municipal Zoo is located in Guarulhos, São Paulo, Brazil; CRC (Callitrichid Research Center) is located in Omaha, Nebraska, US; NEPRC (New England Primate Research Center, no longer in operation) was located in Southborough, Massachusetts, US; CPRJ (Centro de Primatologia do Rio de Janeiro) is located in Guapimirim, Rio de Janeiro, Brazil; CEMAFAUNA (Centro de Concervação e Manejo de Fauna da Caatinga) is located in Petrolina, Pernambuco. Abbreviations for Brazilian states in the 'Sampling Location' column are as follows: Espírito Santo (ES), Minas Gerais (MG), Rio de Janeiro (RJ), São Paulo (SP). DF is the Brazilian Federal District. NA = No data Available.

Sample	Accession Number	Phenotype	mtDNA lineage	Sampling location	Latitude/Longitude
BJT6	MN787074	*C. aurita*	*C. aurita*	Guiricema, MG	-21.010, -42.720
BJT20	MN787075	*C. aurita*	*C. jacchus*	Mogi das Cruzes, SP (Removed from wild and housed at Guarulhos Municipal Zoo)	-23.523, -46.179
BJT22	MZ027648	*C. aurita*	*C. penicillata*	São José dos Campos, SP (Removed from wild and housed at Guarulhos Municipal Zoo)	-23.523, -46.179
BJT65	MT041703	*C. aurita*	*C. aurita*	Guiricema, MG	-21.010, -42.720
BJT79	MN787077	*C. aurita*	*C. jacchus*	Guarulhos Municipal Zoo	NA
BJT83	MN787078	*C. aurita*	*C. aurita*	Sao Jose dos Campos, SP (Removed from wild and housed at Guarulhos Municipal Zoo)	-23.070, -45.933
BJT84	MN787079	*C. aurita*	*C. jacchus*	Guarulhos Municipal Zoo	NA
BJT107	MN787080	*C. aurita*	*C. aurita*	Natividade, RJ (Removed from wild and housed at CPRJ)	-21.043, -41.978
BJT109	MN787081	*C. aurita*	*C. aurita*	Natividade, RJ (Removed from wild and housed at CPRJ)	-21.043, -41.978
BJT114	MN787082	*C. aurita*	*C. aurita*	Guapirim, RJ (Removed from wild and housed at CPRJ)	-22.488, -42.912
Cgeoffroyi_NC_021	NC_021941	*C. geoffroyi*	*C. geoffroyi*	NA	NA
cgeo006	LR745201	*C. geoffroyi*	*C. geoffroyi*	CRC	NA
BJT102	MN787083	*C. geoffroyi*	*C. geoffroyi*	CPRJ	NA
BJT143	MN787084	*C. geoffroyi*	*C. geoffroyi*	Berilo, MG	-16.953, -42.466
BJT169	MN787086	*C. geoffroyi*	*C. geoffroyi*	Serra, ES	-20.213, -40.267
BJT170	MN787085	*C. geoffroyi*	*C. geoffroyi*	Serra, ES	-20.213, -40.267
BJT171	MN787087	*C. geoffroyi*	*C. geoffroyi*	Serra, ES	-20.213, -40.267
cja007	LR745200	*C. jacchus*	*C. jacchus*	NEPRC	NA
Cjacchus KM588314A	KM588314	*C. jacchus*	*C. jacchus*	NA	NA
Cjacchus NC_025586	NC_025586	*C. jacchus*	*C. jacchus*	NA	NA
BJT86	MN787088	*C. jacchus*	*C. jacchus*	Guarulhos Municipal Zoo	NA
BJT87	MN787089	*C. jacchus*	*C. jacchus*	Guarulhos Municipal Zoo	NA
BJT100	MN787090	*C. jacchus*	*C. jacchus*	CPRJ	NA
BJT157	MN787091	*C. jacchus*	*C. jacchus*	CEMAFAUNA	NA
BJT165	MN787092	*C. jacchus*	*C. jacchus*	CEMAFAUNA	NA
BJT166	MN787093	*C. jacchus*	*C. jacchus*	CEMAFAUNA	NA
BJT167	MN787094	*C. jacchus*	*C. jacchus*	CEMAFAUNA	NA
cpe025	MN787101	*C. penicillata*	*C. penicillata*	SWPW Quadra 15, Conjunto 5, Brasília, DF	-15.911, -47.953
cpe030	MN787102	*C. penicillata*	*C. penicillata*	Jardim Botânico, Brasília, DF	-15.861, -47.829
BJT8	MN787095	*C. penicillata*	*C. penicillata*	Lavras, MG	-21.227, -44.980
BJT10	MN787096	*C. penicillata*	*C. penicillata*	Lavras, MG	-21.227, -44.980
BJT40	MN787097	*C. penicillata*	*C. penicillata*	Belo Horizonte, MG	-19.921, -43.990
BJT41	MN787098	*C. penicillata*	*C. penicillata*	Belo Horizonte, MG	-19.921, -43.990
BJT159	MN787099	*C. penicillata*	*C. penicillata*	CEMAFAUNA	NA
BJT160	MN787100	*C. penicillata*	*C. penicillata*	CEMAFAUNA	NA
cku004	KR817257	*C. kuhlii*	*C. kuhlii*	CRC	NA
Ckuhlili KR869628	KR869628	*C. kuhlii*	*C. kuhlii*	NA	NA
Ckuhlii NC_027658	NC_027658	*C. kuhlii*	*C. kuhlii*	NA	NA
BJT24	MN787103	*C. aurita* x sp.	*C. aurita*	Guarulhos Municipal Zoo (apprehended animal)	NA
BJT25	MN787104	*C. aurita* x sp.	*C. jacchus*	Guarulhos Municipal Zoo (apprehended animal)	NA
BJT26	MN787105	*C. aurita* x sp.	*C. penicillata*	Guarulhos Municipal Zoo (apprehended animal)	NA
BJT27	MN787106	*C. aurita* x sp.	*C. geoffroyi*	Guarulhos Municipal Zoo (apprehended animal)	NA
BJT115	MN787107	*C. aurita* x sp.	*C. aurita*	Guapirim, RJ	-22.488, -42.912
BJT116	MN787108	*C. penicillata* x *C. jacchus*	*C. penicillata*	Guapirim, RJ	-22.488, -42.912
BJT70	MN787109	*Callithrix* sp. x *Callithrix* sp.	*C. geoffroyi*	Santa Teresa, ES	-19.935, -40.596
BJT13	MN787110	*C. penicillata* x *C. geoffroyi* (D)	*C. penicillata*	Viçosa, MG	-20.755, -42.872
BJT14	MN787120	*C. penicillata* x *C. geoffroyi* (B)	*C. penicillata*	Viçosa, MG	-20.755, -42.872
BJT15	MN787117	*C. penicillata* x *C. geoffroyi* (D)	*C. penicillata*	Viçosa, MG	-20.755, -42.872
BJT16	MN787114	*C. penicillata* x *C. geoffroyi*	*C. penicillata*	Viçosa, MG	-20.755, -42.872
BJT31	MN787111	*C. penicillata* x *C. geoffroyi* (C)	*C. penicillata*	Viçosa, MG	-20.758, -42.860
BJT33	MN787112	*C. penicillata* x *C. geoffroyi* (B)	*C. penicillata*	Viçosa, MG	-20.778, -42.863
BJT38	MN787113	*C. penicillata* x *C. geoffroyi* (A)	*C. penicillata*	Viçosa, MG	-20.755, -42.857
BJT39	MN787116	*C. penicillata* x *C. geoffroyi* (D)	*C. penicillata*	Viçosa, MG	-20.755, -42.857
BJT75	MN787115	*C. penicillata* x *C. geoffroyi*	*C. penicillata*	Viçosa, MG	-20.759, -42.866
BJT150	MN787118	*C. penicillata* x *C. geoffroyi* (A)	*C. penicillata*	Viçosa, MG	-20.759, -42.866
BJT151	MN787119	*C. penicillata* x *C. geoffroyi* (A)	*C. penicillata*	Viçosa, MG	-20.759, -42.866
Ateles belzebuth	KC757386	NA	NA	NA	NA
Alouatta caraya	KC757384	NA	NA	NA	NA
Aotus azarai	KC757385	NA	NA	NA	NA
Aotus lemurinus	FJ785421	NA	NA	NA	NA
Aotus nancymaae	NC_018116	NA	NA	NA	NA
Brachyteles arachnoides	JX262672	NA	NA	NA	NA
Cacajao calvus	KC959985	NA	NA	NA	NA
Cebuella pygmaea	KC757389	NA	NA	NA	NA
Cebus albifrons	NC_002763	NA	NA	NA	NA
Chiropotes albinasus	KC757393	NA	NA	NA	NA
Lagothrix lagotricha	KC757398	NA	NA	NA	NA
Leontopithecus rosalia	KC757399	NA	NA	NA	NA
Plecturocebus cupreus	KC959986	NA	NA	NA	NA
Plecturocebus donacophilus	FJ785423	NA	NA	NA	NA
Saguinus oedipus	KC757409	NA	NA	NA	NA
Saimiri boliviensis boliviensis	NC_018096	NA	NA	NA	NA
Saimiri oerstedii citrinellus	HQ644336	NA	NA	NA	NA
Saimiri sciureus macrodon	HQ644338	NA	NA	NA	NA
Saimiri oerstedii oerstedii	HQ644337	NA	NA	NA	NA
Saimiri boliviensis peruviensis	HQ644340	NA	NA	NA	NA
Saimiri sciureus sciureus	HQ644334	NA	NA	NA	NA
Sapajus apella	NC_016666	NA	NA	NA	NA
Sapajus xanthosternos	KC757410	NA	NA	NA	NA

References

1. Malukiewicz, J. *et al.* Mitogenomic phylogeny of *Callithrix* with special focus on human transferred taxa. *BMC Genomics* **22**, DOI: 10.1186/s12864-021-07533-1 (2021).

2. Fuzessy, L. F. *et al.* Morphological variation in wild marmosets (callithrix penicillata and c. geoffroyi) and their hybrids. *Evol. Biol.* **41**, 480–493, DOI: 10.1007/s11692-014-9284-5 (2014).
